# Poly[[[aqua(2,2′-bipyridine-κ^2^ *N*,*N*′)zinc(II)]-μ-2-nitroterephthalato-κ^2^ *O* ^1^:*O* ^4^] monohydrate]

**DOI:** 10.1107/S1600536810023615

**Published:** 2010-07-21

**Authors:** Hui-Min Li, Hong-Bo Yuan, Shi-Yao Yang, Rong-Bin Huang

**Affiliations:** a Department of Chemistry, College of Chemistry and Chemical Engineering, Xiamen University, Xiamen 361005, People’s Republic of China; b Shandong Polytechnic Vocational College, Jining 272017, People’s Republic of China

## Abstract

In the title compound, {[Zn(C_8_H_3_NO_6_)(C_10_H_8_N_2_)(H_2_O)]·H_2_O}_*n*_, the Zn^II^ ion is square-pyramidally coordinated, and bridged by 2-*nitro*-terephthalate ligands, forming a chain running along [1
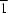
0]. Intra­molecular hydrogen bonds are formed between the coordinated water mol­ecules and the nitro O atoms. Adjacent chains are linked by hydrogen bonds between the coordinated water mol­ecules and the O atoms of the monodentate carboxyl groups.

## Related literature

Benzene polycarb­oxy­lic acids and nitro­gen hetero aromatic ligands have been used to construct Zn^II^ coordination polymers by hydro­thermal synthesis, see: Huang *et al.* (2008 ▶); Ma *et al.* (2005 ▶); Song *et al.* (2006 ▶); Wang *et al.* (2005 ▶); Yang *et al.* (2002 ▶, 2003*a* ▶,*b* ▶,*c* ▶); Zhang *et al.* (2003 ▶, 2007 ▶); Zhou *et al.* (2009*a* ▶) The substituents on the benzene polycarb­oxy­lic acids have been found to play important roles in determining the structures of the coordination polymers, see: Prajapati *et al.* (2009 ▶); Zhou *et al.* (2009*b* ▶).
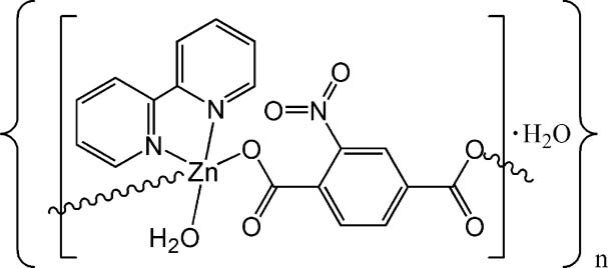


## Experimental

### 

#### Crystal data

[Zn(C_8_H_3_NO_6_)(C_10_H_8_N_2_)(H_2_O)]·H_2_O
*M* *_r_* = 466.70
Triclinic, 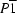

*a* = 8.5570 (5) Å
*b* = 9.1074 (5) Å
*c* = 12.2060 (7) Å
α = 84.558 (1)°
β = 76.863 (1)°
γ = 73.692 (1)°
*V* = 888.58 (9) Å^3^
*Z* = 2
Mo *K*α radiation
μ = 1.44 mm^−1^
*T* = 297 K
0.41 × 0.36 × 0.33 mm

#### Data collection

Bruker SMART APEX area-detector diffractometer
Absorption correction: multi-scan (*SADABS*; Bruker, 2002 ▶) *T*_min_ = 0.590, *T*_max_ = 0.648
5326 measured reflections
3915 independent reflections
3739 reflections with *I* > 2σ(*I*)
*R*_int_ = 0.028

#### Refinement

*R*[*F* ^2^ > 2σ(*F* ^2^)] = 0.041
*wR*(*F* ^2^) = 0.119
*S* = 1.10
3915 reflections
285 parameters
4 restraints
H atoms treated by a mixture of independent and constrained refinement
Δρ_max_ = 0.65 e Å^−3^
Δρ_min_ = −0.39 e Å^−3^

### 

Data collection: *SMART* (Bruker, 2002 ▶); cell refinement: *SAINT* (Bruker, 2001 ▶); data reduction: *SAINT*; program(s) used to solve structure: *SHELXS97* (Sheldrick, 2008 ▶); program(s) used to refine structure: *SHELXL97* (Sheldrick, 2008 ▶); molecular graphics: *ORTEPII* (Johnson, 1976 ▶); software used to prepare material for publication: *SHELXL97*.

## Supplementary Material

Crystal structure: contains datablocks I, global. DOI: 10.1107/S1600536810023615/kp2266sup1.cif

Structure factors: contains datablocks I. DOI: 10.1107/S1600536810023615/kp2266Isup2.hkl

Additional supplementary materials:  crystallographic information; 3D view; checkCIF report

## Figures and Tables

**Table:** 

Zn1—O1	1.9922 (19)
Zn1—O1*W*	2.063 (2)
Zn1—O4^i^	1.976 (2)
Zn1—N1	2.141 (2)
Zn1—N2	2.091 (2)

**Table:** 

O1—Zn1—O1*W*	95.05 (8)
O1—Zn1—N1	90.59 (8)
O1—Zn1—N2	98.69 (9)
O1*W*—Zn1—N1	170.66 (9)
O1*W*—Zn1—N2	94.61 (9)
O4^i^—Zn1—O1	149.63 (10)
O4^i^—Zn1—O1*W*	89.11 (9)
O4^i^—Zn1—N1	89.79 (9)
O4^i^—Zn1—N2	110.97 (10)
N1—Zn1—N2	77.14 (9)

Symmetry code: (i) 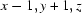.

**Table 2:** Hydrogen-bond geometry (Å, °)

*D*—H⋯*A*	*D*—H	H⋯*A*	*D*⋯*A*	*D*—H⋯*A*
O1*W*—H1*A*⋯O2^ii^	0.85 (3)	1.83 (3)	2.670 (3)	174 (4)
O1*W*—H1*B*⋯O3^iii^	0.84 (3)	2.02 (2)	2.779 (3)	150 (3)
O1*W*—H1*B*⋯O5	0.84 (3)	2.58 (3)	3.031 (3)	115 (3)
O2*W*—H2*A*⋯O2	0.85 (3)	2.03 (3)	2.865 (4)	171 (5)
O2*W*—H2*B*⋯O1^iv^	0.85 (3)	2.57 (4)	3.213 (4)	134 (4)

Symmetry codes: (ii) 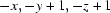; (iii) 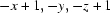; (iv) 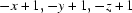.

**Fig. 1.**
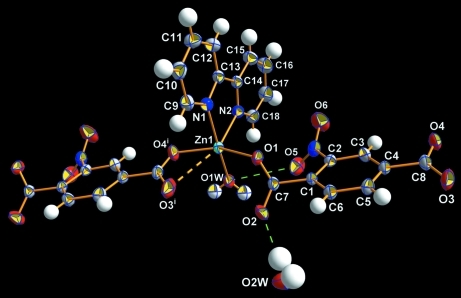
The coordination environment of zinc ion in I with the atom labeling scheme. Ellipsoids are drawn at the 50% probability level. Hydrogen bonds are showm in green dashed line. Symmetry codes: (i) x - 1, y + 1, z.

**Fig. 2.**
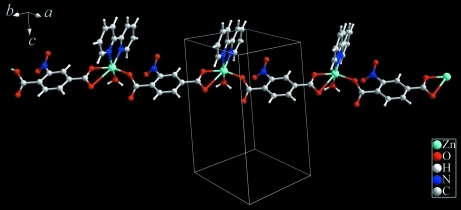
A perspective view of the one-dimensional chain of I.

## supplementary crystallographic information

### Comment

Benzne polycarboxylic acids and nitrogen hetero aromatic ligands have been used to construct Zn^II^ coordination polymers by hydrothermal syntheses. (Huang *et al.*, 2008; Ma *et al.*, 2005; Song *et al.*, 2006; Wang *et al.*, 2005; Yang *et al.*, 2002; Yang *et al.*, 2003*a*,*b*,*c*; Zhang *et al.*, 2007; Zhang *et al.*, 2003; Zhou *et al.*, 2009a) In some of the researches, the substituents on the benzne polycarboxylic acids have been found to play important roles in determining the structures of the coordination polymers (Prajapati *et al.*, 2009; Zhou *et al.*, 2009b) In this paper, we would like to report a coordination polymer, {[Zn(ntp)(H_2_O)(2,2'-bpy)](H_2_O)}_n_, 1 (ntp = 2-*nitro*-terephthalate, 2,2'-bpy = 2,2'-bipyridine) synthesized by hydrothermal reaction.

In the structure of I, the asymmetric unit contains one Zn^II^ ion, one ntp ligand, one coordinated water molecule, one 2,2'-bpy and one solvent water molecule. (Fig. 1, Table 1) The Zn^II^ ion is in a distorted square pyramidal geometry, coordinated by two carboxylate oxygen atoms from two ntp briding ligands, one oxygen atom from water molecule and two nitrogen atoms from 2,2'-bpy. In ntp, the carboxyl in the ortho position of nitro substituent adopts monodentate coordination mode, and the dihedral angle between it and the benzene ring is 45.96 °; the other carboxyl adopts semi-chelating mode, the dihedral angle is 11.35 °. In the semi-chelating mode, one of the coordination bond is very long and weak and is almost neglectable. (Zn1-O3^i^ = 2.859 Å, ^i^*x* - 1, *y* + 1, *z*) The Zn^II^ ion is bridged by ntp ligands to form a one dimensional chain running along [1 -1 0] direction (Fig. 2). Intramolecular hydrogen bonds are formed between the coordinated water molecules and the nitro oxygen atoms. Adjacent chains also form intermolecular hydrogen bonds between the coordinated water molecules and the oxygen atoms of the monodentate carboxyl groups (Table 2).

### Experimental

The suspension of 2-*nitro*-terephthalic acid (0.042 g, 0.20 mmol) and 2,2'-bipyridine (0.033 g, 0.20 mmol) in H_2_O (10 mL) was vigorously stirred, aqueous solution of sodium hydroxide (2 mol/L) was slowly added until the pH value was adjusted to 7, and then ZnCl_2_ (0.027 g, 0.20 mmol) was added. The solution was placed in a 20 mL Teflon-lined vessel, heated to 453 K at the rate of 0.2 K/min, and kept at 453 K for 3 days, and then slowly cooled down to room temperature at the rate of 0.1 K/min. Yellow block crystals (0.035 g, yield 38%) were separated by filtration, washed with deionized water and dried in air. Elemental Analysis: C_18_H_15_N_3_O_8_Zn, found (calc.) C 47.23 (46.32), H 3.30 (3.24), N 9.18 (9.00).

### Refinement

The position of the water H atom were refined with O–H distance restrained to 0.85 Å, with their temperature factors set to 1.2 times those of the parent atoms. The aromatic H atoms were generated geometrically (C–H 0.93 Å) and were allowed to ride on their parent atoms in the riding model approximations, with their temperature factors set to 1.2 times those of the parent atoms.

### Crystal data

**Table:**

[Zn(C_8_H_3_NO_6_)(C_10_H_8_N_2_)(H_2_O)]·H_2_O	*Z* = 2
*M**_r_* = 466.70	*F*(000) = 476
Triclinic, *P*1	*D*_x_ = 1.744 Mg m^−^^3^
Hall symbol: -P 1	Mo *K*α radiation, λ = 0.71073 Å
*a* = 8.5570 (5) Å	Cell parameters from 4213 reflections
*b* = 9.1074 (5) Å	θ = 2.3–28.5°
*c* = 12.2060 (7) Å	µ = 1.44 mm^−^^1^
α = 84.558 (1)°	*T* = 297 K
β = 76.863 (1)°	Block, yellow
γ = 73.692 (1)°	0.41 × 0.36 × 0.33 mm
*V* = 888.58 (9) Å^3^	

### Data collection

**Table:**

Bruker SMART APEX area-detector diffractometer	3915 independent reflections
Radiation source: fine-focus sealed tube	3739 reflections with *I* > 2σ(*I*)
graphite	*R*_int_ = 0.028
φ and ω scan	θ_max_ = 28.6°, θ_min_ = 1.7°
Absorption correction: multi-scan (*SADABS*; Bruker, 2002)	*h* = −10→10
*T*_min_ = 0.590, *T*_max_ = 0.648	*k* = −11→11
5326 measured reflections	*l* = −15→13

### Refinement

**Table:**

Refinement on *F*^2^	Primary atom site location: structure-invariant direct methods
Least-squares matrix: full	Secondary atom site location: difference Fourier map
*R*[*F*^2^ > 2σ(*F*^2^)] = 0.041	Hydrogen site location: inferred from neighbouring sites
*wR*(*F*^2^) = 0.119	H atoms treated by a mixture of independent and constrained refinement
*S* = 1.10	*w* = 1/[σ^2^(*F*_o_^2^) + (0.0588*P*)^2^ + 0.302*P*] where *P* = (*F*_o_^2^ + 2*F*_c_^2^)/3
3915 reflections	(Δ/σ)_max_ = 0.001
285 parameters	Δρ_max_ = 0.65 e Å^−^^3^
4 restraints	Δρ_min_ = −0.39 e Å^−^^3^

### Special details

**Table:**

Geometry. All esds (except the esd in the dihedral angle between two l.s. planes) are estimated using the full covariance matrix. The cell esds are taken into account individually in the estimation of esds in distances, angles and torsion angles; correlations between esds in cell parameters are only used when they are defined by crystal symmetry. An approximate (isotropic) treatment of cell esds is used for estimating esds involving l.s. planes.
Refinement. Refinement of F^2^ against ALL reflections. The weighted R-factor wR and goodness of fit S are based on F^2^, conventional R-factors R are based on F, with F set to zero for negative F^2^. The threshold expression of F^2^ > 2sigma(F^2^) is used only for calculating R-factors(gt) etc. and is not relevant to the choice of reflections for refinement. R-factors based on F^2^ are statistically about twice as large as those based on F, and R- factors based on ALL data will be even larger.

### Fractional atomic coordinates and isotropic or equivalent isotropic displacement parameters (Å^2^)

**Table:**

	*x*	*y*	*z*	*U*_iso_*/*U*_eq_	
Zn1	0.10197 (3)	0.50965 (3)	0.27688 (2)	0.02770 (12)	
O1	0.3177 (2)	0.3927 (2)	0.31591 (17)	0.0375 (4)	
O1W	−0.0424 (3)	0.4071 (2)	0.40216 (18)	0.0373 (4)	
H1A	−0.131 (3)	0.463 (3)	0.440 (3)	0.043 (10)*	
H1B	−0.003 (4)	0.340 (3)	0.448 (2)	0.043 (10)*	
O2	0.3290 (3)	0.4075 (3)	0.49422 (19)	0.0493 (6)	
O2W	0.4501 (5)	0.3808 (4)	0.6976 (2)	0.0700 (8)	
H2A	0.425 (6)	0.386 (6)	0.634 (2)	0.084*	
H2B	0.547 (3)	0.392 (6)	0.673 (4)	0.084*	
O3	1.0362 (3)	−0.2228 (3)	0.4031 (2)	0.0547 (6)	
O4	0.9149 (3)	−0.3030 (2)	0.2882 (2)	0.0460 (5)	
O5	0.2308 (3)	0.1110 (3)	0.3586 (2)	0.0519 (6)	
O6	0.3909 (4)	0.0225 (4)	0.2029 (3)	0.0690 (8)	
N1	0.2312 (3)	0.6052 (2)	0.12874 (19)	0.0302 (4)	
N2	0.0999 (3)	0.3715 (2)	0.15004 (19)	0.0306 (5)	
N3	0.3663 (3)	0.0691 (3)	0.2970 (2)	0.0368 (5)	
C1	0.5136 (3)	0.2006 (3)	0.3915 (2)	0.0258 (5)	
C2	0.5128 (3)	0.0739 (3)	0.3376 (2)	0.0274 (5)	
C3	0.6443 (3)	−0.0554 (3)	0.3223 (2)	0.0317 (5)	
H3A	0.6396	−0.1378	0.2847	0.038*	
C4	0.7833 (3)	−0.0609 (3)	0.3636 (2)	0.0304 (5)	
C5	0.7895 (3)	0.0643 (3)	0.4159 (2)	0.0343 (6)	
H5A	0.8840	0.0619	0.4422	0.041*	
C6	0.6559 (3)	0.1934 (3)	0.4294 (2)	0.0318 (5)	
H6A	0.6621	0.2771	0.4648	0.038*	
C7	0.3725 (3)	0.3455 (3)	0.4030 (2)	0.0283 (5)	
C8	0.9233 (4)	−0.2058 (3)	0.3517 (2)	0.0376 (6)	
C9	0.2869 (4)	0.7290 (3)	0.1244 (3)	0.0378 (6)	
H9A	0.2710	0.7795	0.1904	0.045*	
C10	0.3665 (4)	0.7837 (3)	0.0258 (3)	0.0440 (7)	
H10A	0.4041	0.8703	0.0248	0.053*	
C11	0.3905 (4)	0.7090 (4)	−0.0723 (3)	0.0448 (7)	
H11A	0.4457	0.7438	−0.1402	0.054*	
C12	0.3314 (4)	0.5822 (3)	−0.0682 (2)	0.0395 (6)	
H12A	0.3454	0.5303	−0.1333	0.047*	
C13	0.2514 (3)	0.5336 (3)	0.0338 (2)	0.0302 (5)	
C14	0.1825 (3)	0.3991 (3)	0.0464 (2)	0.0306 (5)	
C15	0.1997 (4)	0.3087 (4)	−0.0424 (3)	0.0438 (7)	
H15A	0.2559	0.3304	−0.1140	0.053*	
C16	0.1324 (5)	0.1859 (4)	−0.0232 (3)	0.0510 (8)	
H16A	0.1440	0.1225	−0.0816	0.061*	
C17	0.0489 (4)	0.1579 (4)	0.0818 (3)	0.0464 (7)	
H17A	0.0020	0.0757	0.0960	0.056*	
C18	0.0344 (4)	0.2527 (3)	0.1669 (3)	0.0389 (6)	
H18A	−0.0231	0.2332	0.2387	0.047*	

### Atomic displacement parameters (Å^2^)

**Table:**

	*U*^11^	*U*^22^	*U*^33^	*U*^12^	*U*^13^	*U*^23^
Zn1	0.02659 (18)	0.02438 (17)	0.02802 (18)	0.00097 (12)	−0.00622 (12)	−0.00332 (11)
O1	0.0309 (10)	0.0370 (10)	0.0351 (10)	0.0057 (8)	−0.0081 (8)	0.0037 (8)
O1W	0.0307 (10)	0.0347 (10)	0.0358 (11)	0.0018 (8)	0.0006 (8)	0.0006 (8)
O2	0.0432 (12)	0.0515 (12)	0.0397 (12)	0.0159 (10)	−0.0096 (10)	−0.0196 (10)
O2W	0.096 (2)	0.088 (2)	0.0453 (15)	−0.0528 (19)	−0.0214 (15)	0.0056 (14)
O3	0.0361 (12)	0.0516 (13)	0.0633 (16)	0.0094 (10)	−0.0150 (11)	0.0090 (11)
O4	0.0421 (12)	0.0311 (10)	0.0485 (13)	0.0097 (9)	0.0000 (10)	−0.0035 (9)
O5	0.0312 (11)	0.0619 (14)	0.0611 (15)	−0.0123 (10)	−0.0113 (10)	0.0092 (12)
O6	0.0621 (17)	0.0779 (18)	0.0722 (18)	−0.0005 (14)	−0.0334 (15)	−0.0364 (15)
N1	0.0317 (11)	0.0271 (10)	0.0298 (11)	−0.0043 (8)	−0.0068 (9)	−0.0017 (8)
N2	0.0302 (11)	0.0288 (10)	0.0323 (11)	−0.0032 (8)	−0.0101 (9)	−0.0032 (8)
N3	0.0367 (13)	0.0285 (11)	0.0477 (14)	−0.0062 (9)	−0.0173 (11)	0.0000 (10)
C1	0.0237 (11)	0.0238 (10)	0.0248 (11)	0.0002 (9)	−0.0033 (9)	0.0002 (9)
C2	0.0265 (12)	0.0262 (11)	0.0287 (12)	−0.0051 (9)	−0.0073 (9)	0.0015 (9)
C3	0.0350 (14)	0.0240 (11)	0.0329 (13)	−0.0027 (10)	−0.0055 (11)	−0.0049 (9)
C4	0.0285 (12)	0.0254 (11)	0.0293 (12)	0.0010 (9)	−0.0016 (10)	0.0027 (9)
C5	0.0259 (12)	0.0378 (13)	0.0365 (14)	−0.0012 (10)	−0.0106 (11)	0.0001 (11)
C6	0.0300 (13)	0.0291 (12)	0.0356 (14)	−0.0027 (10)	−0.0103 (11)	−0.0057 (10)
C7	0.0232 (11)	0.0246 (11)	0.0319 (13)	−0.0003 (9)	−0.0028 (10)	−0.0009 (9)
C8	0.0333 (14)	0.0281 (12)	0.0368 (15)	0.0039 (11)	0.0036 (11)	0.0064 (11)
C9	0.0405 (15)	0.0309 (13)	0.0417 (16)	−0.0078 (11)	−0.0087 (12)	−0.0047 (11)
C10	0.0459 (17)	0.0334 (14)	0.0508 (18)	−0.0131 (13)	−0.0053 (14)	0.0033 (12)
C11	0.0468 (17)	0.0478 (17)	0.0371 (16)	−0.0145 (14)	−0.0038 (13)	0.0054 (13)
C12	0.0451 (16)	0.0413 (15)	0.0301 (14)	−0.0093 (12)	−0.0055 (12)	−0.0035 (11)
C13	0.0278 (12)	0.0287 (11)	0.0310 (13)	−0.0008 (10)	−0.0074 (10)	−0.0037 (10)
C14	0.0297 (12)	0.0288 (11)	0.0317 (13)	−0.0017 (10)	−0.0096 (10)	−0.0039 (10)
C15	0.0501 (18)	0.0452 (16)	0.0371 (15)	−0.0095 (14)	−0.0110 (13)	−0.0125 (13)
C16	0.063 (2)	0.0447 (17)	0.053 (2)	−0.0159 (16)	−0.0197 (17)	−0.0158 (14)
C17	0.0522 (19)	0.0402 (15)	0.0542 (19)	−0.0177 (14)	−0.0179 (15)	−0.0055 (14)
C18	0.0406 (15)	0.0386 (14)	0.0385 (15)	−0.0108 (12)	−0.0105 (12)	0.0001 (12)

### Geometric parameters (Å, °)

**Table:**

Zn1—O1	1.9922 (19)	C3—C4	1.381 (4)
Zn1—O1W	2.063 (2)	C3—H3A	0.9300
Zn1—O4^i^	1.976 (2)	C4—C5	1.377 (4)
Zn1—N1	2.141 (2)	C4—C8	1.506 (3)
Zn1—N2	2.091 (2)	C5—C6	1.383 (4)
O1—C7	1.249 (3)	C5—H5A	0.9300
O1W—H1A	0.85 (3)	C6—H6A	0.9300
O1W—H1B	0.84 (3)	C9—C10	1.366 (4)
O2—C7	1.230 (3)	C9—H9A	0.9300
O2W—H2A	0.85 (3)	C10—C11	1.379 (5)
O2W—H2B	0.85 (3)	C10—H10A	0.9300
O3—C8	1.234 (4)	C11—C12	1.377 (4)
O4—C8	1.257 (4)	C11—H11A	0.9300
O4—Zn1^ii^	1.976 (2)	C12—C13	1.376 (4)
O5—N3	1.210 (3)	C12—H12A	0.9300
O6—N3	1.215 (3)	C13—C14	1.483 (4)
N1—C9	1.334 (4)	C14—C15	1.380 (4)
N1—C13	1.337 (3)	C15—C16	1.374 (5)
N2—C18	1.332 (4)	C15—H15A	0.9300
N2—C14	1.340 (4)	C16—C17	1.357 (5)
N3—C2	1.461 (3)	C16—H16A	0.9300
C1—C6	1.381 (4)	C17—C18	1.375 (4)
C1—C2	1.384 (3)	C17—H17A	0.9300
C1—C7	1.510 (3)	C18—H18A	0.9300
C2—C3	1.375 (3)		
			
O1—Zn1—O1W	95.05 (8)	C1—C6—C5	121.3 (2)
O1—Zn1—N1	90.59 (8)	C1—C6—H6A	119.3
O1—Zn1—N2	98.69 (9)	C5—C6—H6A	119.3
O1W—Zn1—N1	170.66 (9)	O2—C7—O1	127.0 (2)
O1W—Zn1—N2	94.61 (9)	O2—C7—C1	117.3 (2)
O4^i^—Zn1—O1	149.63 (10)	O1—C7—C1	115.6 (2)
O4^i^—Zn1—O1W	89.11 (9)	O3—C8—O4	124.5 (3)
O4^i^—Zn1—N1	89.79 (9)	O3—C8—C4	119.8 (3)
O4^i^—Zn1—N2	110.97 (10)	O4—C8—C4	115.8 (3)
N1—Zn1—N2	77.14 (9)	N1—C9—C10	121.9 (3)
C7—O1—Zn1	137.57 (18)	N1—C9—H9A	119.1
Zn1—O1W—H1A	118 (2)	C10—C9—H9A	119.1
Zn1—O1W—H1B	124 (2)	C9—C10—C11	119.2 (3)
H1A—O1W—H1B	106 (3)	C9—C10—H10A	120.4
H2A—O2W—H2B	96 (5)	C11—C10—H10A	120.4
C8—O4—Zn1^ii^	113.6 (2)	C12—C11—C10	119.0 (3)
C9—N1—C13	119.3 (2)	C12—C11—H11A	120.5
C9—N1—Zn1	125.6 (2)	C10—C11—H11A	120.5
C13—N1—Zn1	115.06 (17)	C13—C12—C11	118.9 (3)
C18—N2—C14	118.6 (2)	C13—C12—H12A	120.6
C18—N2—Zn1	124.7 (2)	C11—C12—H12A	120.6
C14—N2—Zn1	116.48 (18)	N1—C13—C12	121.7 (3)
O5—N3—O6	124.9 (3)	N1—C13—C14	115.5 (2)
O5—N3—C2	118.3 (3)	C12—C13—C14	122.8 (2)
O6—N3—C2	116.7 (3)	N2—C14—C15	121.7 (3)
C6—C1—C2	116.8 (2)	N2—C14—C13	115.6 (2)
C6—C1—C7	120.2 (2)	C15—C14—C13	122.7 (3)
C2—C1—C7	122.9 (2)	C16—C15—C14	118.9 (3)
C3—C2—C1	123.1 (2)	C16—C15—H15A	120.5
C3—C2—N3	116.1 (2)	C14—C15—H15A	120.5
C1—C2—N3	120.8 (2)	C17—C16—C15	119.4 (3)
C2—C3—C4	118.9 (2)	C17—C16—H16A	120.3
C2—C3—H3A	120.6	C15—C16—H16A	120.3
C4—C3—H3A	120.6	C16—C17—C18	119.2 (3)
C5—C4—C3	119.6 (2)	C16—C17—H17A	120.4
C5—C4—C8	121.8 (3)	C18—C17—H17A	120.4
C3—C4—C8	118.6 (3)	N2—C18—C17	122.3 (3)
C4—C5—C6	120.4 (3)	N2—C18—H18A	118.9
C4—C5—H5A	119.8	C17—C18—H18A	118.9
C6—C5—H5A	119.8		
			
O4^i^—Zn1—O1—C7	58.0 (4)	C6—C1—C7—O2	−46.1 (4)
O1W—Zn1—O1—C7	−38.9 (3)	C2—C1—C7—O2	137.9 (3)
N2—Zn1—O1—C7	−134.3 (3)	C6—C1—C7—O1	130.8 (3)
N1—Zn1—O1—C7	148.6 (3)	C2—C1—C7—O1	−45.1 (3)
O4^i^—Zn1—N1—C9	64.9 (2)	Zn1^ii^—O4—C8—O3	0.0 (4)
O1—Zn1—N1—C9	−84.8 (2)	Zn1^ii^—O4—C8—C4	−179.80 (17)
N2—Zn1—N1—C9	176.5 (2)	C5—C4—C8—O3	10.4 (4)
O4^i^—Zn1—N1—C13	−113.31 (19)	C3—C4—C8—O3	−168.5 (3)
O1—Zn1—N1—C13	97.06 (19)	C5—C4—C8—O4	−169.8 (3)
N2—Zn1—N1—C13	−1.72 (18)	C3—C4—C8—O4	11.3 (4)
O4^i^—Zn1—N2—C18	−96.8 (2)	C13—N1—C9—C10	−1.0 (4)
O1—Zn1—N2—C18	89.8 (2)	Zn1—N1—C9—C10	−179.1 (2)
O1W—Zn1—N2—C18	−6.0 (2)	N1—C9—C10—C11	−0.1 (5)
N1—Zn1—N2—C18	178.4 (2)	C9—C10—C11—C12	0.8 (5)
O4^i^—Zn1—N2—C14	88.66 (19)	C10—C11—C12—C13	−0.4 (5)
O1—Zn1—N2—C14	−84.70 (19)	C9—N1—C13—C12	1.4 (4)
O1W—Zn1—N2—C14	179.47 (18)	Zn1—N1—C13—C12	179.7 (2)
N1—Zn1—N2—C14	3.91 (18)	C9—N1—C13—C14	−178.8 (2)
C6—C1—C2—C3	0.8 (4)	Zn1—N1—C13—C14	−0.5 (3)
C7—C1—C2—C3	176.8 (2)	C11—C12—C13—N1	−0.7 (4)
C6—C1—C2—N3	178.4 (2)	C11—C12—C13—C14	179.5 (3)
C7—C1—C2—N3	−5.5 (4)	C18—N2—C14—C15	0.5 (4)
O5—N3—C2—C3	132.7 (3)	Zn1—N2—C14—C15	175.4 (2)
O6—N3—C2—C3	−47.2 (4)	C18—N2—C14—C13	179.7 (2)
O5—N3—C2—C1	−45.1 (3)	Zn1—N2—C14—C13	−5.4 (3)
O6—N3—C2—C1	135.0 (3)	N1—C13—C14—N2	3.8 (3)
C1—C2—C3—C4	0.8 (4)	C12—C13—C14—N2	−176.4 (2)
N3—C2—C3—C4	−176.9 (2)	N1—C13—C14—C15	−176.9 (3)
C2—C3—C4—C5	−2.0 (4)	C12—C13—C14—C15	2.9 (4)
C2—C3—C4—C8	177.0 (2)	N2—C14—C15—C16	−1.0 (5)
C3—C4—C5—C6	1.5 (4)	C13—C14—C15—C16	179.8 (3)
C8—C4—C5—C6	−177.4 (2)	C14—C15—C16—C17	0.9 (5)
C2—C1—C6—C5	−1.2 (4)	C15—C16—C17—C18	−0.4 (5)
C7—C1—C6—C5	−177.4 (2)	C14—N2—C18—C17	0.1 (4)
C4—C5—C6—C1	0.1 (4)	Zn1—N2—C18—C17	−174.4 (2)
Zn1—O1—C7—O2	−33.2 (5)	C16—C17—C18—N2	−0.1 (5)
Zn1—O1—C7—C1	150.2 (2)		

Symmetry codes: (i) *x*−1, *y*+1, *z*; (ii) *x*+1, *y*−1, *z*.

### Hydrogen-bond geometry (Å, °)

**Table:**

*D*—H···*A*	*D*—H	H···*A*	*D*···*A*	*D*—H···*A*
O1W—H1A···O2^iii^	0.85 (3)	1.83 (3)	2.670 (3)	174 (4)
O1W—H1B···O3^iv^	0.84 (3)	2.02 (2)	2.779 (3)	150 (3)
O1W—H1B···O5	0.84 (3)	2.58 (3)	3.031 (3)	115 (3)
O2W—H2A···O2	0.85 (3)	2.03 (3)	2.865 (4)	171 (5)
O2W—H2B···O1^v^	0.85 (3)	2.57 (4)	3.213 (4)	134 (4)

Symmetry codes: (iii) −*x*, −*y*+1, −*z*+1; (iv) −*x*+1, −*y*, −*z*+1; (v) −*x*+1, −*y*+1, −*z*+1.
